# Validation of verbal autopsy: determination of cause of deaths in Malaysia 2013

**DOI:** 10.1186/s12889-017-4668-y

**Published:** 2017-08-11

**Authors:** Shubash Shander Ganapathy, Yi Yi Khoo, Mohd Omar Azahadi, Mohamad Anuar Mohamad Fuad, Chandrika Jeevananthan, Chalapati Rao

**Affiliations:** 1 Institut Kesihatan Umum (Institute of Public Health), Ministry of Health Malaysia, Jalan Bangsar, 50590 Kuala Lumpur, Malaysia; 2 Department of Social and Preventive Medicine, University of Malaya, Kuala Lumpur, Malaysia; 3 Department of Global Health, Research School of Population Health, Australian National University, Canberra, Australia

**Keywords:** Verbal autopsy, Validation, Mortality, Cause of death

## Abstract

**Background:**

Mortality statistics by age, sex and cause are the foundation of basic health data required for health status assessment, epidemiological research and formation of health policy. Close to half the deaths in Malaysia occur outside a health facility, are not attended by medical personnel, and are given a lay opinion as to the cause of death, leading to poor quality of data from vital registration. Verbal autopsy (VA) is a very useful tool in diagnosing broad causes of deaths for events that occur outside health facilities. This article reports the development of the VA methods and our principal finding from a validation study.

**Methods:**

A cross sectional study on nationally representative sample deaths that occurred in Malaysia during 2013 was used. A VA questionnaire suitable for local use was developed. Trained field interviewers visited the family members of the deceased at their homes and conducted face to face interviews with the next of kin. Completed questionnaires were reviewed by trained physicians who assigned multiple and underlying causes. Reference diagnoses for validation were obtained from review of medical records (MR) available for a sample of the overall study deaths.

**Results:**

Corresponding MR diagnosis with matched sample of the VA diagnosis were available in 2172 cases for the validation study. Sensitivity scores were good (>75%) for transport accidents and certain cancers. Moderate sensitivity (50% - 75%) was obtained for ischaemic heart disease (64%) and cerebrovascular disease (72%). The validation sample for deaths due to major causes such as ischaemic heart disease, pneumonia, breast cancer and transport accidents show low cause-specific mortality fraction (CSMF) changes. The scores obtained for the top 10 leading site-specific cancers ranged from average to good.

**Conclusion:**

We can conclude that VA is suitable for implementation for deaths outside the health facilities in Malaysia. This would reduce ill-defined mortality causes in vital registration data, and yield more accurate national mortality statistics.

## Background

Mortality statistics by age, sex and cause are the foundation and the most basic health data of a country. With the advent of improved health policies and research, appropriate assignment of cause of death and a reliable death registration is vital for health status assessment, epidemiological research and formation of health policy. However, very few countries appear to have complete coverage of death registration and high quality data [1]. The quality of vital statistics is especially poor in the African and Asian regions, with minimal progress seen globally since the new millennium [2].

Malaysia has maintained a functional vital registration for many decades. Deaths in Malaysia are broadly classified to 2 main categories – medically certified deaths and non-medically certified deaths. Medically certified deaths are deaths occurring in health facilities or attended by a medical officer. Based on the presenting symptoms and examination, the cause of death is determined by the medical officer. Non-medically certified deaths on the other hand occur outside a health facility. The cause of death is recorded as determined by a lay person, often a police officer or the next of kin of the deceased. Due to the lack of medical attention, the cause of death recorded is often symptomatic, ill-defined and unreliable.

Data released by Department of Statistics Malaysia reported that the non-medically certified deaths account for 47.9% of the total deaths in 2013 for Malaysia, with over 60% of these uncertified deaths listing “Old Age” as the cause of death [3]. This serves to highlight the extent of uncertainty in the current Malaysia mortality statistics. Proportion of ill-defined causes of death are the best indicator of the quality of national cause-of-death data and poor quality data does not provide reliable information for national health policy development [4].

In order to improve the quality of the mortality statistics in the country, the Ministry of Health Malaysia had undertaken to implement a nationally representative study utilizing Verbal Autopsy (VA) methods adapted for the Malaysian context to ascertain the cause of death that occurred outside hospitals in 2013. VA methods have been used in many countries around the world. Even though VA assessment was initially used in small settings, research on VA methods have improved and simplified it for population level use and integration at national level [5].

VA has been identified as a very useful tool in diagnosing broad causes of death that occur outside health facilities and where physicians are not present to certify all causes of registered deaths [6]. A qualitative study had shown that verbal autopsy has good acceptability and support within the communities in Malaysia [7]. It is also a cost effective tool in providing information to guide policy and set priorities in countries undergoing rapid mortality transitions in the absence of a complete civil registry system [8]. However it is essential to validate VA methods within a population to understand the performance characteristics of the VA instrument and address any potential bias that arise, such as systematic over-estimation and under-estimation of deaths from particular causes [9]. This article reports the development of the VA methods and our principal finding from the validation study.

## Methods

### Study design and objectives

A cross sectional study on nationally representative sample deaths that occurred in Malaysia during 2013 was used to ascertain causes of death using Verbal Autopsy (VA) methods. This study is part of a larger research project to test methodologies in a national sample of deaths to verify registered causes for hospital deaths, and determine causes for community deaths in Malaysia. The overall objective of the larger research project is to develop more reliable estimates of cause-specific mortality in Malaysia, and the complete methodology is published elsewhere [10]. The objectives of this component of the project were to adapt international VA standards for application in Malaysia and for verification and validation of the locally adapted VA method.

### Local adaptation of VA methods

VA methods have previously never been used in Malaysia. To develop a VA method suitable for local use, we reviewed international VA questionnaires and adapted these to the Malaysian setting. Various expert groups from each medical field discussed and developed consensus on the structure and content of each specific item within the questionnaire. The VA questionnaire was developed for two specific age groups; below 12 years and 12 years and above. An open narrative component together with closed questions was designed to allow chronological narratives to supplement the structured methods [11]. The broad content structure of the questionnaires is summarized in Table 1 below. The adapted questionnaires were subsequently translated to Bahasa Malaysia, the local language and prepared in dual language, Bahasa Malaysia and English. The preliminary adapted VA version and all related study material were pilot tested on a sample of 50 medically certified deaths and 50 uncertified deaths prior to the study. Both the pilot test studies for medically certified deaths and uncertified deaths were conducted between January 2014 and February 2014 in Kajang, Selangor. Any modifications, as suggested from the pilot studies, were carried out, for the final version of the study materials.

**Table 1:** Content Structure of Malaysia Verbal Autopsy (VA) Questionnaire

Deaths Below 12 Year-Old	Items	Deaths Above 12 Year-Old	Items
Open Ended Narration	1	Open Ended Narration	1
Background	25	Injury / Accident	5
Maternal History	16	Symptoms Checklist	91
Neonatal Death (Age < 28 days)	48	Question for Female	15
Infant and Child Death	49	Alcohol and Tobacco	9
Health Record	19	History of Chronic Disease	26
		Health Records	15
TOTAL ITEMS	158	TOTAL ITEMS	162

Upon review, the information in the hospital medical records of those deceased in health facilities was extracted into a prepared “Medical Record Abstraction Form”. This form documented the key clinical notes, laboratory and radiological findings, surgical notes and treatment history in specifically designed separate sections of the form, to ensure that all relevant clinical characteristics of the case are adequately documented and captured in the abstraction form to aid the determination of cause of death.

### Sampling plan

The estimated total sample size for the overall research project was calculated for the mortality measurement across the range of demographic and epidemiological scenarios in Malaysia as required for mortality surveillance [12]. A population sample of 11,000 deaths was required for measurement of cause-specific mortality rates by 5 year age-sex groups according to three broad cause groups (communicable diseases, non-communicable diseases and injuries). The primary sampling units were the districts in Malaysia, with probability proportionate to size approach used to select the districts for the study. Deaths in 2012 were used for sample size calculation with assumption that the death in 2013 would bear similar characteristics. The sample size is further inflated by 1.25 to take into account the design effect, and a further 10% for dropout during the study due to loss to follow up. Thus a final sample size of 15,000 deaths was determined for this research project.

All the 144 districts across Malaysia were stratified by the number of deaths in 2012 into three strata: districts with less than 500 deaths; those with 500 to 1000 deaths; and those with more than 1000 deaths. Subsequently, using probability proportionate to size approach, 19 districts were selected by stratified random sampling to adequately cover the calculated sample size. The 19 districts consists of 4 districts with more than 1000 deaths, 10 districts with 500 to 1000 deaths,and 5 districts with less than 500 deaths. All deaths that occurred in the selected districts during the study reference year (2013) were included in the study sample. The characteristics of the total deaths from the selected sample of districts were also tested and found to be adequately representative at the national level in terms of age group, gender and proportion of hospital and non-hospital deaths.

### Field implementation

Prior to field implementation of the VA, training programs were conducted for all staff and personnel involved in the;

1. Verbal Autopsy Interviews
2. Medical Record Abstraction
3. Physician Death Certification from VA Questionnaire / Medical Records Abstraction
4. ICD Coding of causes of death and selection of the underlying cause
5. Study administration including field coordination, supervision and data management

The National Registration Department (NRD) provided a list of all deaths with details of address, reporting institution and cause of death at registration for these selected districts for the year 2013. The list of deaths was then forwarded to the selected District Health Office (DHO). The cause of death in the registration was blinded to the DHO and field interviewers to ensure the interview is free of bias from this aspect.

For each of the death in the list;

- Trained field interviewers visited the family members of the deceased at their homes and conducted face to face interviews with a family member or the individual who was closest to the deceased and present with the deceased during their illness preceding the death. Informed written consent was taken before each interview. Consent was also obtained from the interviewee to review the medical records of the deceased for deaths that had occurred in a medical facility.
- All completed questionnaires were returned to the DHO where it was submitted to teams of trained physicians consisting of public health specialists and family medicine specialists to assign causes of death based on the format required in international medical death certificates. Each questionnaire was reviewed by one physician, who may seek second opinion when necessary in assigning the probable causes of death.
- All deaths in health facilities were concurrently followed up by an independent trained team to review the medical records of the deceased and for abstraction of information into study forms.
- The completed medical records abstraction forms were sent to a panel of medical specialists. One physician, from a trained panel of medical specialists, who may seek a second opinion when necessary, reviewed and ascertained the causes of death using the international medical death certificate. The panel of medical experts had been trained on death certification and were provided with diagnostic guidelines for major disease causes in order to ensure uniformity and accuracy of diagnosis [13].
- Trained coders reviewed all completed death certificates from the VA and medical records abstraction to assign cause of death consistent with the *International Classification of Disease and Related Health Problems, tenth revision* (ICD-10) codes and rules to determine the underlying cause of death in 3 characters ICD Code [14].

Adequate quality control measures were also placed throughout the data collection and processing, using field supervisors to manually verify completeness of the questionnaires and consistency of responses. The field supervisors from the districts and health facilities also acted as the liaison officers to the research team. We used one physician to determine the cause of death as previous studies have shown substantial agreement in assigning cause of death for nationally represented sample when 2 independent physicians are used [15]. A central team of experts from the Institute of Public Health Malaysia also reviewed the death certificates and ICD codes. Independence of the VA team and the medical records abstraction team, together with training provided to public health specialists, family medicine specialists and physicians involved in the study for causes of death assignments ensured high degree of concurrence in certification practice and reliability of cause of death ascertainment.

Data collection was conducted between March 2014 and December 2015, resulting in VA recall period ranging from 3 months to 3 years. All data from VA interview, medical records abstraction, death certificate, ICD Codes and underlying cause of death was entered into a database for analysis.

### Data analysis

The underlying cause of death was designated to one of the 103 cause category based upon the World Health Organization (WHO) Mortality Tabulation List 1 for all further analysis. The validation of the VA method used for deaths in Malaysia was determined by comparing the VA diagnosis with review of medical records (MR) diagnosis across these 103 groups. The agreement of the VA, assignment to other causes and assigned from other causes compared to MR was determined for each group. The validity of the VA was assessed by sensitivity, specificity and positive predictive value with the MR cause of death as the reference standard. The cause-specific mortality fraction (CSMF) for each cause of death was also determined. Local sensitivity, specificity and CSMF are essential for cause of death estimation as they differ substantially by populations with different patterns of cause specific mortality [16].

## Results

Reference or gold standard diagnosis obtained from review of medical records (MR) from a sample of deaths in hospitals was used to validate the verbal autopsy (VA). The initial study sample of 14,497 deaths included 7487 hospital deaths, but of these, MR diagnosis was obtained for 5988 deaths (80.0%). MR diagnosis could not be obtained for the remaining hospital deaths as the written case files could not be found. Also, for this sample of hospital deaths, field operations were successful in completing VA interviews in only 3013 cases (40%). Finally, corresponding MR diagnosis with matched VA diagnosis for the validation analyses were available in only 2172 cases of the total sample of hospital deaths (29%).

**Fig. 1:**
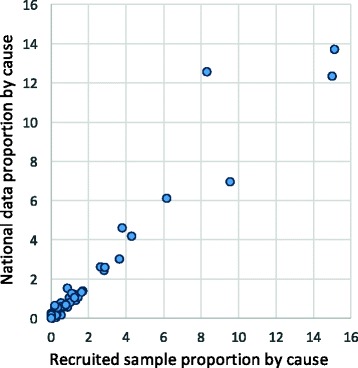
Comparison of proportional cause distributions from national data and final study sample of hospital deaths, 2013

The VA response rate for each district ranged between 24.1% to 55.0%. The losses to follow up was analysed to determine if it resulted in any major biases in the final study sample. Figure 1 shows a scatter plot of proportion of specific cause for all hospital deaths in Malaysia on the y axis, and its corresponding proportion as per registration diagnosis for the successfully recruited sample on the x axis. The majority of cause proportions by the specific cause category in the study population and the study sample are found to be similar, falling along the 45° line. The successful study sample had a smaller representation of ill-defined cause compared to the national population with 8.3% to 12.6%. The concordance between the two distributions suggest that despite the large losses to follow up, the study sample is largely non-differential by cause compared to the national population.

Table 2 below presents the findings for the VA validation results. Sensitivity scores were good (>75%) for transport accidents and certain cancers. This indicates that if VA diagnoses a death to be from these causes, it is likely to be accurate. Moderate sensitivity (50% - 75%) was obtained for other leading causes of death such as ischaemic heart disease (64%) and cerebrovascular disease (72%). On the other hand, sensitivity scores were poor (<50%) for pneumonia (37%), diabetes (36%) and chronic lower respiratory diseases (48%). The impact of poor sensitivity scores may be minimized by compensatory misclassification patterns in the sample. The deaths due to major causes such as ischaemic heart disease, pneumonia, breast cancer and transport accidents show low cause-specific mortality fraction (CSMF) changes. This indicates compensatory misclassification patterns are present for these causes. Overall, VA tends to overdiagnose diseases of the liver, chronic lower respiratory diseases, respiratory tuberculosis and colorectal cancer and underdiagnose renal failure and cerebrovascular disease.

**Table 2:** Validation characteristics of Verbal Autopsy (VA) procedures for 15 leading causes of hospital deaths

Cause of deaths	Medical Record (MR) diagnosis	Verbal Autopsy (VA) diagnosis	Validation scores for VA		
			Sensitivity (95% CI)	PPV	CSMF changes in VA (%)^a^
Ischaemic heart diseases	311	316	0.65 (0.60, 0.71)	0.64	1.6
Cerebrovascular diseases	282	236	0.60 (0.55, 0.66)	0.72	−16.3
Transport accidents	193	206	0.92 (0.88, 0.96)	0.86	6.7
Pneumonia	182	178	0.37 (0.30, 0.44)	0.38	−2.2
Diabetes mellitus	91	102	0.36 (0.26,0.46)	0.32	12.1
Chronic lower respiratory diseases	73	99	0.48 (0.36, 0.59)	0.35	35.6
Other heart diseases	48	65	0.21 (0.09, 0.32)	0.15	35.4
Renal failure	80	64	0.35 (0.25, 0.45)	0.44	−20.0
Other diseases of the digestive system	58	42	0.33 (0.21, 0.45)	0.73	−27.6
Other malignant neoplasms	60	41	0.38 (0.26.0.51)	0.45	−31.7
Respiratory tuberculosis	30	40	0.47 (0.29, 0.65)	0.56	33.3
Cancer of colon, rectum and anus	28	37	0.71 (0.55, 0.88)	0.35	32.1
Breast cancer	34	36	0.94 (0.86, 1.00)	0.54	5.9
Diseases of the liver	25	36	0.40 (0.21, 0.59)	0.89	44.0
All other causes	677	674			
Total deaths	2172	2172			

^a^indicates the change in cause-specific mortality fractions (CSMF) from VA

Positive change indicates over diagnosis by VA; Negative change indicates under diagnosis by VA

The matrix table in Table 3 outlines the misclassification patterns for the important causes of death in the study sample that result in the changes to cause-specific mortality proportions. A high number of deaths caused by transport accidents classified by VA were confirmed upon medical review (177/206 deaths). 72% (170/236) of deaths classified to cerebrovascular disease by VA were found to be accurate by MR review. Only 28% of deaths classified by VA to diseases of the liver, 32% to diabetes mellitus and 35% to respiratory tuberculosis were confirmed by MR review. This presents that VA overcounts deaths resulting from these diseases, whereby the number of deaths misdiagnosed to these diseases by VA is more than the number of deaths found to be truly due to these diseases on MR review. This is an important point to note for ischaemic heart disease, that even though the CSMF from VA and MR is low, a considerable portion is misclassified with incorrect diagnosis by VA.

**Table 3:** Discrepancies observed between Verbal Autopsy (VA) diagnosis and Medical Records (MR) review

Cause of deaths	Medical Record (MR) Diagnosis																
Verbal Autopsy (VA) Diagnosis	67	69	96	74	52	76	68	86	34	81	46	5	30	36	80	All other causes	Total
Ischaemic heart diseases (67)	203	24	2	13	10	8	9	7	1	2			3			34	316
Cerebrovascular diseases (69)	8	170	2	13	8	1	3	4		4	1					22	236
Transport accidents (96)	2	2	177	1	1			1								22	206
Pneumonia (74)	9	13	1	67	4	12	5	8	2		3	1			2	51	78
Diabetes mellitus (52)	7	7	1	8	33	4	3	5		3	1	1	1	1		27	102
Chronic lower respiratory diseases (76)	13	10		19	2	35	4	1	2	2	1					10	99
Other heart diseases (68)	18	2		10	3	1	10	1		1					1	18	65
Renal failure (86)	4	1		3	5	1	3	28		1						18	64
Trachea, bronchus and lung cancer (34)		1		3	1	1			44		2	2				6	60
Other diseases of the digestive system (81)	1	1		3		1	1	2		19		1				13	42
Other malignant neoplasms (46)	2			2	1				1		23	1	2			9	41
Respiratory tuberculosis (5)	2	3		8	1	1						14				11	40
Cancer of colon, rectum and anus (30)	1				1				1	1	6		20			7	37
Breast cancer (36)		1									1	1		32	1		36
Diseases of the liver (80)	1	1	1	2	1					4		1			10	15	36
All other causes	40	46	9	30	20	8	10	23	7	21	22	8	2	1	11	356	614
TOTAL	311	282	193	182	91	73	48	80	58	58	60	30	28	34	25	619	2172

We further analysed the utilization of VA to validate deaths caused by cancer. Table 4 below shows the results of the validation of the top 10 leading site-specific cancer deaths in Malaysia. Overall, the sensitivity scores ranged from average to good (58.3–94.1) for the top 10 leading site-specific cancers. VA was found to be very good at identifying deaths due to breast cancer with a very high sensitivity score of 94.1% and 34 out of 36 deaths classified to this by VA were confirmed upon MR review. Cancer of colon, rectum and anus, as well as stomach cancer, had a low PPV of 54% and 43%, indicating overdiagnosis by VA for these causes. On the other hand, deaths from leukaemia, liver, brain and pancreatic cancers were undercounted. However, deaths that were categorized by VA to these cancer causes had high positive predictive value (PPV) scores of 75%, 77%, 88% and 83% respectively.

**Table 4:** Validation characteristics of Verbal Autopsy (VA) procedures for 10 leading site-specific cancer deaths in the study sample

Cause of death	Medical record (MR) diagnoses	Verbal autopsy (VA) diagnoses	Validation scores for VA		
			Sensitivity	PPV	CSMF change in VA (%)^a^
Trachea, bronchus and lung cancer	58	60	75.9	73.3	3.4
Cancer of colon, rectum and anus	28	37	71.4	54.1	32.1
Breast cancer	34	36	94.1	88.9	5.9
Liver cancer	25	22	68.0	77.3	−12.0
Leukaemia	20	16	60.0	75.0	−20.0
Stomach cancer	8	14	75.0	42.9	75.0
Lip, oral cavity and pharyngeal cancer	8	11	75.0	54.5	37.5
Cervix cancer	6	8	83.3	62.5	33.3
Brain cancer	12	8	58.3	87.5	−33.3
Pancreatic cancer	8	6	62.5	83.3	−25.0

^a^indicates the change in cause-specific mortality fractions (CSMF) from VA

Positive change indicates over diagnosis by VA; Negative change indicates under diagnosis by VA

## Discussion

Medical record diagnosis was used as the gold standard for validation of the verbal autopsy (VA). The validation study is essential to understand the misclassification patterns and biases arising from VA procedure in ascertaining cause of death for the Malaysian context. Assumptions are made that the characteristics of the deaths in the hospital sample are applicable for deaths outside health facilities.

For population level validation of verbal autopsy, diagnostic accuracy is considered acceptable if the sensitivity is above 50% and the difference in cause specific mortality fraction (CSMF) is below the 20% threshold [17]. VA diagnoses for important leading causes of death in Malaysia such as transport accidents, ischaemic heart diseases and cerebrovascular diseases, were all found to have good sensitivity in our study. Similar findings were seen for all site-specific cancer deaths. We also found that with exception of a few diseases, most of the major causes of death had CSMF values within the defined acceptable threshold. Although determining the validation and obtaining the sensitivity, specificity and CSMF is essential for a VA study, the assessment of VA should most importantly focus on its usefulness of filling gaps within mortality data to address public health needs [11].

The findings of this study will be the basis for adjusting the estimates derived from applying VA procedures for deaths occurring outside health facilities in Malaysia. Application of VA will not only aid in correcting the raw registration diagnosis for deaths outside health facilities in Malaysia, but also derive a more accurate final estimate of cause-specific mortality by age and sex for deaths in Malaysia. There is a real possibility that those who die in the absence of medical attention may be the ones in most need for health protection and are essential to be included in national health plans and policies, and identification of the causes for such deaths will therefore enable better targeted health policies in Malaysia [18].

We must also keep in mind that the VA would require on-going changes based on feedback from the field operators and periodical analysis to determine more accurate estimations. Further refinement of the questionnaire and estimates would certainly improve the quality of the information derived from VA. It is only logical to expect problems to arise when initiated, that can only be resolved by modifications in local-level operations and further research upon implementation [19]. This study serves as an important initial step in the introduction of VA in Malaysia, and the validation study findings as well as the field experiences provide sufficient justification to scale up the use of these methods in other parts of the country.

Other VA validation studies in developing countries have all shown promising results of using VA for cause of death assignment. A study in Thailand, Malaysia’s neighbour country, in 2005 had similarly shown good sensitivity, specificity and low CSMF change for leading cause of deaths such as transport accidents, ischaemic heart diseases and cerebrovascular diseases [20]. The VA validation study in Tanzania produced good results across a broad range of diseases and disease groups for neonatal, infant and adult mortality [8]. Likewise to our own study, the VA validation study in China had shown that even though there may be poor validity of some individual cause of deaths, compensatory classification that occurs for broad cause of deaths highlights the importance of the validation study and implementing VA for public health utility [21].

With the worldwide focus on Millennium Development Goals now being replaced by the Sustainable Development Goals, priorities in health statistics now include a much wider scope of disease categories, ranging from acute infectious diseases, non-communicable diseases and injuries [22]. In countries experiencing rapid health status changes, such as Malaysia, understanding the population health dynamic and strengthening the national health information system is essential for developing meaningful health policies [23]. As more countries undertake to improve their morality statistics, this would be a step forward from global “estimates”, towards global “measurements” of disease burden [24].

One of the major strengths of this study is that this study was carried out with a large sample that is nationally representative. However, this was offset by the considerably losses to follow up in conducting VAs. Despite the losses to follow up, the study sample was non-differential from the national sample. This study also highlights the need to improve medical record maintenance in Malaysian hospitals as seen by the considerable case files that could not be traced during this study. Another potential limitation of this study is the recall bias by the next of kin of the deceased. Recall bias is generally affected by the length of time between the event and the interview, which in our study was between 1 and 3 years. However, for an event as tragic as a loss of life, the length of time of more than a year and up to 5 years recall has been shown to not affect the data quality [25, 26]. In our study, the major problems resulting in losses to follow up as well as long recall periods were the migration and unavailability of the relatives of the deceased for interview, and these reasons have also been experienced in other settings [27].

We must also take into account that the deceased in this validation study are among those who died in the health facilities. This would influence the information received from the next of kin as they may not be fully aware of the disease symptoms that developed or progressed during the hospitalization or may even choose to not disclose this information. Their responses may also be influenced by information provided by health workers during the hospitalization [21]. In a multi-ethnic society like Malaysia, linguistic and conceptual challenges adds a further dimension to the interviews carried out [28]. However, a validation exercise for the verbal autopsy procedure cannot be practically carried out without these limitations [29]. The quality of the interview has also been shown to affect the reliability and accuracy of diagnosis of individual death [30]. One can reduce this bias by ensuring the interviewers are properly trained to conduct the interview and extract the information from the next of kin. It is essential to note that VA is only intended as an interim method of improved vital statistics estimation until a country is ready for a fully functioning and complete civil registration system [31]. Use of a locally adapted VA methodology, as developed and validated in this study, to support the civil registration based mortality statistics system would improve the empirical basis for mortality measurement.

## Conclusion

We can conclude that VA is suitable for implementation for deaths outside the health facilities in Malaysia. Implementation of VA would reduce ill-defined mortality causes, and yield more accurate national mortality statistics.

## Abbreviations

CI: Confidence interval
CSMF: Cause Specific Mortality Fraction
DHO: District Health Office
ICD: International Classification of Disease and Related Health Problems,
MR: Medical Records
MREC: Medical Research Ethics Committee
NRD: National Registration Department
PPV: Positive Predictive Value
VA: Verbal Autopsy
WHO: World Health Organization
