# Erratum to: Validation of verbal autopsy: determination of cause of deaths in Malaysia 2013

**DOI:** 10.1186/s12889-017-4687-8

**Published:** 2017-08-31

**Authors:** Shubash Shander Ganapathy, Khoo Yi Yi, Mohd Azahadi Omar, Mohamad Fuad Mohamad Anuar, Chandrika Jeevananthan, Chalapati Rao

**Affiliations:** 1 Institut Kesihatan Umum (Institute of Public Health), Ministry of Health Malaysia, Jalan Bangsar, 50590 Kuala Lumpur, Malaysia; 2 Department of Social and Preventive Medicine, University of Malaya, Kuala Lumpur, Malaysia; 3 Department of Global Health, Research School of Population Health, Australian National University, Canberra, Australia

## Erratum

After publication of the article [1], it has been brought to our attention that some of the authors had their names presented incorrectly. Author 2 should be “Khoo Yi Yi”, author 3 should be “Mohd Azahadi Omar” and author 4 should be “Mohamad Fuad Mohamad Anuar”. These corrections have been made in the original version of the article.
